# Molecular characterization of extraintestinal and diarrheagenic *Escherichia coli* blood isolates

**DOI:** 10.1080/21505594.2022.2147735

**Published:** 2022-11-24

**Authors:** Vanstokstraeten R., Crombé F., Piérard D., Castillo Moral A., Wybo I., De Geyter D., Janssen T., Caljon B., Demuyser T.

**Affiliations:** a Department of Microbiology and Infection Control, Vrije Universiteit Brussel (VUB), Universitair Ziekenhuis, Brussels, Belgium; b Vrije Universiteit Brussel (VUB), Universitair Ziekenhuis, Brussels Interuniversity Genomics High Throughput core (BRIGHTcore) platform, Brussels, Belgium

**Keywords:** *Escherichia coli*, virulence factors, pathotypes, sequence type, serotype, nosocomial, core genome sequencing, bacteraemia, MLST, hybrid pathotype

## Abstract

Pathogenic *E. coli* strains can be classified into two major groups, based on the presence of specific virulence factors: extraintestinal pathogenic *E. coli* (ExPEC) and diarrheagenic *E. coli* (DEC). Several case reports describe that DEC can cause bloodstream infections in some rare cases. This mainly concerns a few specific sequence types that express virulence factors from both ExPEC and DEC. In this study, we retrospectively analysed 234 *E. coli* blood isolates with whole genome sequencing (WGS). WGS was performed on an Illumina NovaSeq6000. Genotyping was performed using BioNumerics software. The presence of genes was determined with a minimum percentage sequence identity (ID) threshold of 95% and a minimum length for sequence coverage of 95%. Three of the 234 (1.28%) isolates were defined as DEC, 182 (77.78%) as ExPEC, and 49 (20.94%) did not carry pathotype-associated virulence genes. We identified 112 different virulence genes, 48 O-antigens, and 28 H-antigens 82 STs, among the 234 analyzed isolates. ST131 and ST88 were related to healthcare-associated infections. This study provides insight into the prevalence of virulence factors in a large set of *E. coli* blood isolates from the UZ Brussel. It illustrates high diversity in virulence profiles and highlights the potential of DEC to carry virulence factors associated with extraintestinal infections, making it possible for unusual pathotypes to invade and survive in the bloodstream causing bacteraemia. Diarrheagenic strains causing bacteremia are rare and presently underreported, but modern sequencing techniques will better underscore their importance.

## Introduction

*Escherichia coli (E. coli)* is part of the commensal flora of the human intestine and rarely causes disease in healthy individuals. Nonetheless, there is a wide variety of pathogenic *E. coli* strains able to cause diarrhoea or extraintestinal infections. Based on the presence of specific virulence factors, we can classify pathogenic *E. coli* into two major groups: extraintestinal pathogenic *E. coli* (ExPEC) and diarrheagenic *E. coli* (DEC) [1].

ExPEC strains can invade, survive and cause infection in tissues and fluids outside of the intestinal tract, typically the bloodstream and urinary tract. Two major pathotypes within this group are uropathogenic *E. coli* (UPEC) and meningitis- and sepsis-associated *E. coli* (MNEC). UPEC is the most common cause of both complicated and uncomplicated urinary tract infections through a set of specific virulence factors that aid in migration, adhesion, and persistence within the urinary tract. Type 1 pili, like the *fimH* mannose-binding pili, play a central role in the initial attachment to the epithelial cells of the urethra [2]. Sequence type (ST) 131 is of particular interest, which is an emerging and virulent multidrug-resistant strain predominantly isolated from the urinary tract and bloodstream infections [3]. MNEC is the leading cause of early-onset meningitis, accounting for more than 30% of neonatal infections. The *K1* antigen is a shared characteristic among most MNEC serotypes, which promotes serum resistance, intracellular survival, and traversal of the blood-brain barrier [2].

DEC strains can lead to a plethora of diarrhoeal syndromes and are usually not identified outside the intestine. DEC expresses adhesins, invasins, and cytotoxins that target the gastro-intestinal epithelium causing osmotic dysregulation or cell damage. There are five major types of DEC: Shiga toxin-producing *E. coli* (STEC), enterotoxigenic *E. coli* (ETEC), enteropathogenic *E. coli* (EPEC), enteroinvasive *E. coli* (EIEC), and enteroaggregative *E. coli* (EAEC). All STEC isolates encode *stx1* and/or *stx2*, which are potent toxins that target endothelial cells in the gastrointestinal tract and renal glomeruli. ETEC strains are characterized by the production of heat-labile and/or heat-stable enterotoxins, which can dysregulate multiple ion channels in intestinal epithelial cells leading to fluid loss. The LEE-encoded virulence factors *eae* and *tir* are two virulence factors that can induce lesions within intestinal epithelial cells, characteristic for EPEC and part of STEC, qualified as “typical.” EIEC has a close relationship with *Shigella*, both carrying the pINV plasmid harbouring multiple genes like *set2, sigA*, and *ipaH* responsible for cellular fluid loss leading to watery diarrhea. Finally, EAEC is a heterogeneous group characterized by a “stacked brick” architecture and the presence of typical virulence factors like *aggR* and *aaiC* [4].

Virulence genes are often transmissible between *E. coli* strains and are localized on chromosomes, plasmids, or phages. Horizontal gene transfer is probably the main driver for virulence and genetic diversity. It enables the transformation of non-pathogenic *E. coli* to specific pathotypes [5]. Pathotypes are defined by the host’s clinical symptoms and a small set of pathotype-associated virulence genes [6].

However, it should be mentioned that the plasticity of the *E. coli* genome has led to more virulent hybrid pathogenic strains carrying virulence factors of different pathotypes, making it sometimes possible for DEC strains to cause extraintestinal infections, mainly urinary tract infections [7]. Reports of DEC strains causing bacteraemia are very rare. Because routine processing of blood isolates does not address genotyping, these hybrid strains inducing bacteremia are probably strongly underreported. Modern techniques such as whole genome sequencing (WGS) could help us to better identify these particular cases and provides information on the spread of specific bacterial clones responsible for bloodstream infections.

In the current research paper, we aimed to gain an insight into the prevalence of virulence factors present in *E. coli* causing bacteraemia in a relatively large set of samples collected between 1985 and 2018 in the University Hospital of Brussels. Based on these virulence factors, we aimed to look if bloodstream infections with hybrid or atypical pathotypes were occurring in our hospital. As relatively little is known about the clonal structure of *E. coli* causing bacteremia in Belgium, we also described sequence type (ST) using the Achtman typing scheme [8] and compared core genome multilocus sequence type (cgMLST) data. Special attention was paid to a subgroup of 130 isolates for which a medical record was available, where an attempt was made to link ST to the source of infection (healthcare-acquired or community-acquired). Unusual or interesting cases are discussed in more detail with relevant additional data.

## Material and methods

The UZ Brussel is a tertiary care centre with over 700 beds. Two hundred and thirty-four (234) non-duplicate clinical isolates were investigated. These *E. coli* strains were isolated from clinical blood samples randomly selected between 1985 and 2018. All isolates were stored at −80°C. The included isolates are distributed over the years as follows: 1985 (n = 10), 1990 (n = 11), 1995 (n = 12), 2000 (n = 34), 2005 (n = 33), 2010 (n = 33), 2015 (n = 51), and 2018 (n = 50).

The genomic DNA for whole genome sequencing was extracted using the Dneasy blood & tissue kit (Qiagen, Hilden, Germany) for 30 samples and DNA libraries were prepared via the KAPA Hyper Plus kit (Kapa Biosystems, Wilmington, MA, USA). All libraries were sequenced on a MiSeq instrument (Illumina, San Diego, CA, USA) using the v2 (2 × 250 bp) and v3 (2 × 300 bp) reagent kits. Of the other 203 samples, genomic DNA was extracted using the Maxwell RSC Cell DNA purification kit (Promega Corporation, Madison, USA). Fragmentation of 500 ng of genomic DNA was carried out using the NEBNext® Ultra™ II FS module. Sequencing libraries, with an insert size of on average 550 bp, were prepared using the KAPA Hyper Plus kit (Kapa Biosystems, Wilmington, USA) and a Pippin Prep (Sage Science, Beverly, MA, USA) size with the CDF1510 1.5% agarose dye-free cassette selection. To avoid PCR bias, the PCR amplifications step was omitted and a 500 ng input of genomic DNA was used. After equimolar pooling, libraries were sequenced on a Novaseq 6000 instrument (Illumina, San Diego, CA, USA) using the NovaSeq 6000 SP Reagent Kit (500 cycles) generating 2 × 250 bp reads. For this, the library was denatured and diluted according to the manufacturer’s instructions. A 1% PhiX control library was included in each sequencing run.

De novo assembly was performed using SPAdes genome assembler in BioNumerics v.8.1 (Applied Maths, BioMérieux, Sint-Martens-Latem, Belgium).

The *E. coli* functional genotyping plugin version 2.1 incorporated in BioNumerics v.8.1 was used to predict *E. coli* pathotypes, serotypes and virulence gene profiles (194 putative virulence genes; Blast minimum sequence identity 95%, Blast minimum length for coverage 95%).

Assembled genomes were analysed using both the MLST Achtman scheme (7 MLST loci) and the core EnteroBase scheme (2.506 core loci) for *E. coli*/*Shigella* available in BioNumerics v.8.1. The assembly-based approach was used for allele calling. Minimal spanning trees (MSTs) were generated using the MLST Achtman allelic profiles and the core Enterobase allelic profiles as input data in BioNumerics. Branch lengths reflect the number of allele differences (AD) between the isolates in the connected nodes. For cgMLST clustering, the AD threshold was set at <5.

The quality of the sequence read sets, the *de novo* assemblies, and the assembly-based allele calls was verified using the quality statistics window in BioNumerics v.8.1.

Of 130 of the 234 patients, a medical record was available. These medical records were used to see if the sample collection was taken before (community-acquired infection) or after at least 48 hours of hospitalization (healthcare-acquired infection). A Fisher exact test with an alpha value of 0.05 was used to compare data.

## Results

### Pathotypes

Based on a pathogen-defining algorithm (*Supplementary data 1*), different pathotypes were found. One hundred and seventy-one of the 234 (73.01%) isolates carried one or more ExPEC-associated loci. Of these 171 isolates, 138 (80.07%) carried UPEC-specific loci. Thirteen of the 234 (5.56%) isolates carried UPEC-specific loci without the presence of ExPEC-specific loci. Forty-nine of the 234 (20.94%) isolates did not carry pathotype-associated virulence genes. Three of the 234 (1.28%) isolates carried virulence genes associated with DEC, which is highly unusual for bloodstream infections. These three unusual pathotypes belonged to ST38 (hybrid EAEC/ExPEC/UPEC), ST10 (hybrid EAEC/ExPEC), and ST13128 (EPEC).

#### Clinical and molecular characteristics of the atypical pathotypes

The first hybrid isolate (EAEC/ExPEC/UPEC) was isolated from a 76-year-old man in 2015 and belonged to ST38. The man presented to the emergency department of our hospital in poor condition with melaena, fever, tremor, loss of strength, general malaise, and pain all over the body. Both blood and urine cultures revealed an extended-spectrum beta-lactamase (ESBL) producing *E. coli*. The patient was successfully treated with meropenem. Virulence factors characteristic for UPEC (uropathogenic *Escherichia coli*), ExPEC (extraintestinal pathogenic *Escherichia coli*), and EAEC were identified. The EAEC pathotype-associated locus is *aggR*. The ExPEC pathotype-associated loci are *papC, iutA, papA_F7-2* and *kpsMII*. The UPEC pathotype-associated loci are *fyuA* and *chuA*. Several acquired resistance genes were detected: *mdf(a), dfrA1, aac(6’)-Ib-cr, blaOXA-1, sul2, blaCTX-M-15 and tet(A)* resulting in a resistant phenotype for almost all clinically relevant antibiotics. Mutations were found in *gyrA, parC*, and *parE* leading to resistance to nalidixic acid and ciprofloxacin. The serotype was determined as O153/O178:H30.

The second hybrid isolate (EAEC/ExPEC) isolate was isolated from a one-year-old boy in 1990 and belonged to ST10. No further clinical information was available. Virulence factors characteristic of both EAEC and ExPEC were identified. The EAEC pathotype-associated locus is *aggR*. The ExPEC-associated loci are *papC, iutA, papsA_fsiA_F16*, and *kpsMII*. The acquired resistance gene *blaTEM-1B* was detected. No mutational resistance was found. The serotype was determined as O21:H2.

The last unusual pathotype (atypical EPEC) was isolated from a 65-year-old man in 2018 and belonged to the new ST13128. The nearest STs are ST10972, ST7912, ST20, ST3674 and ST561. The man presented to the emergency department of our hospital with abdominal pain. He was known for rectum carcinoma. Due to positive blood cultures, which revealed an *E. coli*, eight days of hospitalization were required. The bacteraemia originated probably from a polyp visualized on a colonoscopy. The patient was successfully treated with amoxicillin/clavulanic acid. Virulence factors characteristic of an atypical EPEC were identified. The atypical EPEC pathotype-associated locus is *eae*. The acquired resistance gene *mdf(A)* conferring resistance to macrolides was detected. No mutational resistance was found. The serotype was determined as O128:H2. All the above-mentioned clinical and functional genotypic characteristics are summarized in *Supplementary data 2*.

### Virulence factors

We identified 112 different virulence genes among the 234 analysed isolates. These genes were classified based on their biochemical properties: adherence, protease, toxin, complement protease, regulation, type 3 translocated protein, survival, type 6 translocated protein, stress protein, secretion system, invasion, antiphagocytosis, and iron uptake in Table 1. Of these 112 virulence factors, 13 (*terC, ompT, iss, sitA, irp2, fyuA, traT, kpsE, chuA, lutA, lucC, papC* and *yfcV*) were detected in 50% or more of the 234 isolates. In contrast, 31 virulence factors were found in 1% or less of these isolates, highlighting high variability. The regulation virulence factor *terC*, responsible for tellurite resistance, was the only virulence factor isolated in all 234 isolates. Adherence-associated virulence factors were the most common: to this group belonged 47 different genes, of which 218 (93.16%) of the 234 isolates had at least one. A detailed list of genes can be found in *Supplementary data 3*.

**Table 1.** Virulence factors detected in the 234 *E. coli* blood isolates.

Pathotypes	All ExPEC (n=182)			Hybrid ExPEC/DEC		DEC	Not defined
Virulence factors	ExPEC (n=32)	ExPEC/UPEC (n=137)	UPEC (n=13)	EAEC/ExPEC (n=1)	EAEC/ExPEC/UPEC (n=1)	EPEC (n=1)	Not specified (n=49)
Adherence (n = 218)	30	137	13	1	1	1	35
Antiphagocytosis (n = 45)	3	36	5	0	0	0	1
Complement protease (n = 162)	32	88	11	0	1	1	29
Invasion (n = 206)	29	135	13	1	0	1	27
Iron uptake (n = 209)	32	137	13	1	1	0	25
Protease (n = 164)	11	127	10	1	1	0	14
Regulation (n = 234)	32	137	13	1	1	1	49
Secretion system (n = 160)	11	135	10	1	1	0	2
Stress protein (n = 87)	0	80	7	0	0	0	0
Survival (n = 193)	30	120	9	1	1	1	31
Toxin (n = 164)	27	111	9	0	0	0	17
Type I translocated protein (n = 65)	20	35	1	0	0	0	9
Type III translocated protein (n = 1)	0	0	0	0	0	1	0
Type VI translocated protein (n = 1)	0	0	0	1	0	0	0

### Multi-locus sequence typing

A total of 82 STs were identified among the 234 isolates when using the Achtman seven gene-MLST scheme. Among these STs, one was not described before (ST13128). Five dominant STs were observed: 26 (11.11%) ST73, 25 (10.68%) ST131, 21 (8.97%) ST69, 18 (7.69%) ST95, and 15 (6.41%) ST88. Eighty-two different O- and 28 different H-serotypes were identified and showed a correlation with ST. For example, 24/25 (96.00%) of the ST131 isolates had serotype O25:H4. Looking at the occurrence of these STs as a function of time, we see that ST131 is significantly (P = 0.0048) more prevalent in the period 2010–2018 (21/100 isolates) than in the period 1985–2005 (4/134 isolates) *(Supplementary Excel file).*

**Figure 1.**
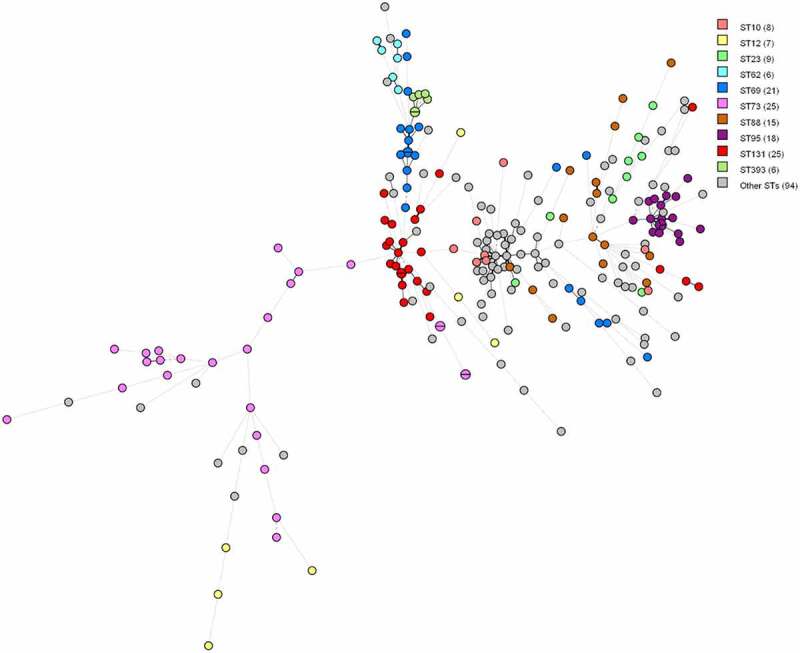
MST based on virulence allelic profiles of the 234 *E. coli* blood isolates. The analysis was carried out in Bionumerics. Nodes are colour-coded per ST. In brackets next to the ST is the number of strains with that ST. Branch length is directly proportional to the allelic difference.

In some cases, the dominant STs and serotypes tend to show a correlation with virulence factor profiles, as they typically harboured very similar sets of virulence genes. Especially isolates within ST393, ST95 and ST62 seem to be very similar based on virulence genes since the nodes are close to each other on the minimum spanning tree (MST) (Figure 1). However, this is not the case for all STs. In isolates within ST12 and ST88, there appears to be little similarity between virulence genes since the nodes are far apart on the MST (Figure 1).

**Figure 2.**
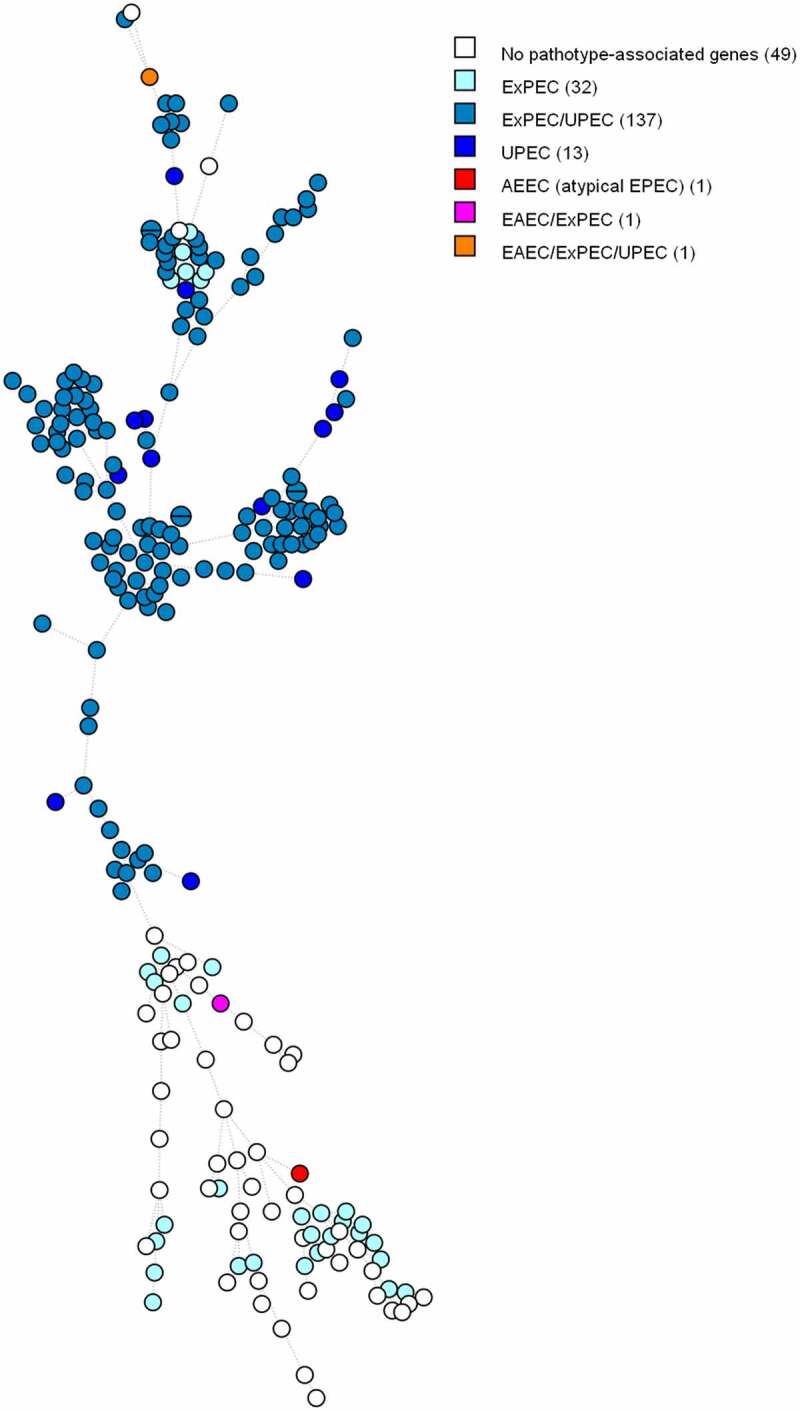
MST based on cgMLST analysis of 234 *E. coli* blood isolates. The analysis was carried out in Bionumerics. Nodes are coloured per pathotype. In brackets next to the pathotype is the number of strains belonging to that pathotype. Branch length is directly proportional to the allelic difference.

Based on the MST tree in Figure 2, we suggest that the pathotype is correlated with the core genome since nodes harbouring identical pathotypes are often positioned close to each other. Except for a few outliers, ExPEC/UPEC and UPEC isolates tend to cluster together. We observe the same trend between ExPECs and isolates without pathotype-associated genes. The three DECs are far apart from each other on the figure, and therefore do not seem to be genetically associated.

#### Healthcare-acquired infections

**Figure 3.**
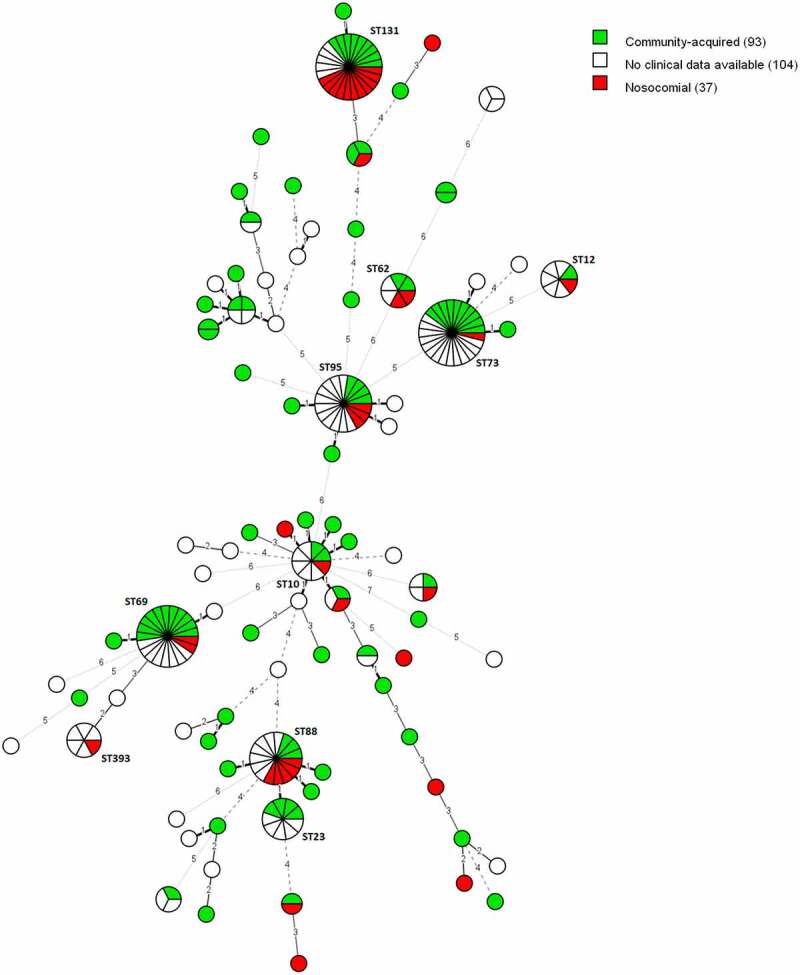
MST of STs using the Achtman seven-gene MLST scheme for 234 *E. coli* blood isolates. The analysis was carried out in Bionumerics. The tree is coloured based on the infection type of the isolates (green: community-acquired, red: healthcare-acquired). Thirty-five nodes are colored red and 93 nodes are colored green. Branch length and width (dotted versus non-dotted) are directly proportional to the allelic difference (ranging from one for the shortest branches to seven for the longest branches).

In the subset of 130 isolates for which a medical record was available, 37 (28.46%) of the positive blood samples were collected for the first time at least 48 hours after hospitalization. These infections are defined as healthcare-acquired. Eleven (29.73%) of these 37 healthcare-acquired infections were caused by ST131 and five (13.51%) by ST88, which caused both significantly (P = 0.0069 and P = 0.0416) more healthcare-acquired infections than community-acquired infections (Figure 3).

#### Clustering

**Figure 4.**
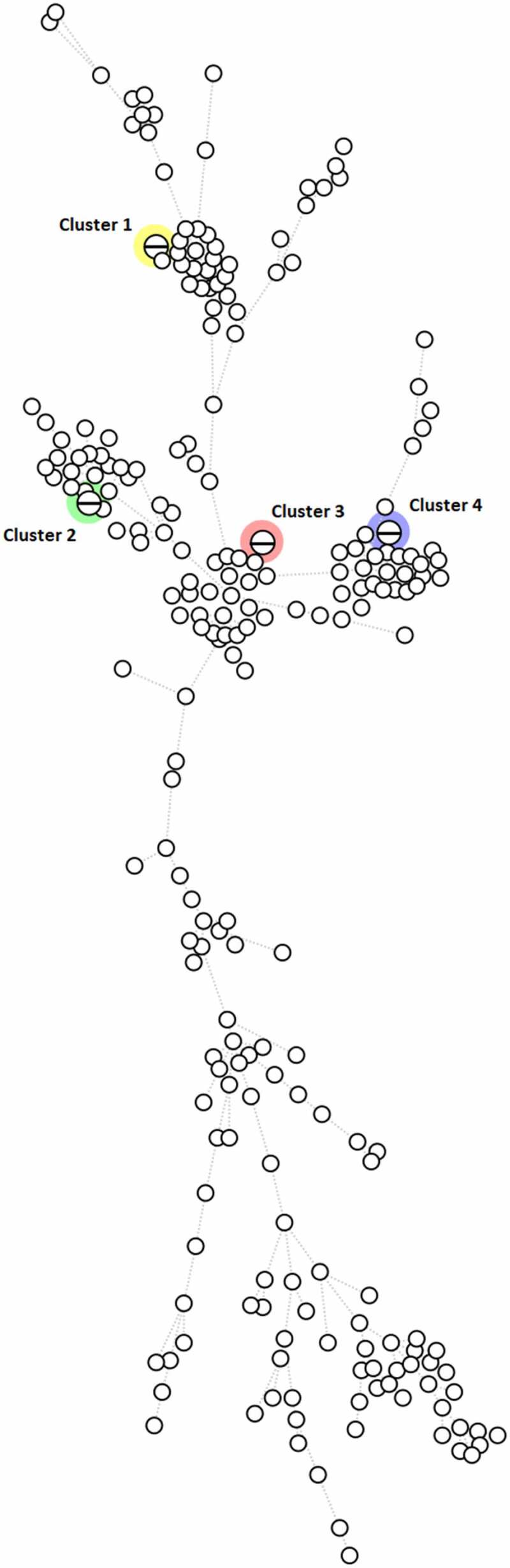
MST based on cgMLST analysis of 234 *E. coli* blood isolates. The analysis was carried out in Bionumerics. A fixed threshold of < 5 allelic differences was used for clustering isolates, highlighting four different clusters. Branch length is directly proportional to the allelic difference.

Four clusters, consisting of two isolates per cluster were identified based on the cgMLST analysis of the 234 *E. coli* blood isolates. (Figure 4).

The two isolates from the first cluster belonged to ST73. Both isolates were isolated from two different patients in September 2005. No further medical data was available.

The two isolates from the second cluster belonged to ST69. The first sample was collected on 8 March 2018 and the second sample on 9 March 2018. However, both samples were taken 48 hours before admission to the hospital, suggesting a community-acquired infection. The clinical focus of the first patient’s bacteraemia was probably the urinary tract, and that of the second patient was probably cholangitis.

The third cluster consisted of two ST95 isolates. The samples were collected on 4 May 2018 and 14 June 2018. In both cases, the urinary tract was the clinical focus of the bacteraemia and the sample was collected within 48 hours of hospitalization. These two patients lived within walking distance of each other. However, it is not clear whether they had direct contact with each other.

The last cluster comprised two ST131 isolates. Both samples were collected on the same date in 2018 within 48 hours of hospitalization, pointing to a healthcare-acquired infection. Both patients were also hospitalized in the same unit, suggesting transmission between the environment and patients or healthcare workers and patients.

## Discussion

The conventional understanding that *E. coli* strains causing diarrhoea are distinct from those that cause extraintestinal disease according to their repertoire of pathotype-associated genes conflicts with our findings. We report a hybrid EAEC/ExPEC ST10, a hybrid EAEC/ExPEC/UPEC ST38, and an atypical EPEC ST13128 causing bacteraemia.

Previous studies already pointed out the potential of both EAEC ST10 and EAEC ST38 to cause extraintestinal infections, mainly urinary tract infections [9,10]. In 2014, Chattaway *et al*. identified eight multidrug-resistant EAEC isolates from urine specimens and one from a blood culture. EAEC ST38 was the most common ST. They described ST38 as an emerging UPEC/EAEC hybrid strain that probably originated from the gut and independently acquired both phenotypes [9]. Alghoribi *et al*. also described ST38 as an evolving multidrug-resistant strain with both EAEC and UPEC virulence factors. They highlight that plasmid-mediated carriage of the EAEC transport regulator gene *aggR* is a key factor of emerging ST38. Accordingly, they screened 15 ST38 isolates for *aggR* and found that four were positive. They noticed no clear differentiation between the amounts of virulence factors present in *aggR*-positive or *aggR*-negative strains [10]. ST10 is one of the most common STs in the *E. coli* MLST database [11]. Olesen *et al*. reported in 2012 an outbreak of a single clonal EAEC ST10 causing 19 urinary tract infections in Copenhagen. They highlighted the potential of ST10 to exhibit both EAEC and ExPEC characteristics. All the ST10 isolates had highly homogeneous virulence profiles. Notably, all outbreak isolates carried one or more of the EAEC pathotype-associated genes: *aggR*, *aatA* and *aaiC*. In contrast, the only detected ExPEC-defining gene was *iutA*. However, the outbreak isolates all contained *fyuA*, *traT*, sat, and *pic*, which are all associated with ExPEC [12]. In our EAEC ST10 case, the strain did not carry virulence genes coding for toxins, yet its enterotoxigenic nature could be related to the acquisition of plasmid-borne toxin-coding virulence factors. Error-prone repair genes on *aggA*-bearing aggregative plasmids, which are harboured by many EAEC ST10 isolates, could play an important role [11].

The MLST of our atypical bacteraemia-related EPEC strain has not been previously described in Enterobase (https://enterobase.warwick.ac.uk/), therefore it was determined as ST13128. The serotype was identified as O128ab:H2, a serotype often related to the EPEC pathotype-associated locus *eae*. In 2013, Buvens *et al*. described a case of a DEC with serotype O128ab:H2 causing bacteremia leading to sepsis. The strain acquisitioned both *stx1* and *st2* genes defining this strain as a STEC. In this case, the infection led to haemolytic-uraemic syndrome (HUS), highlighting the potential of STEC to also cause severe extraintestinal infections [13].

This research showed a high variation in genomic characteristics of *E. coli* strains causing bloodstream infections. The most prevalent genes are *terC, ompT, iss, sitA, irp2* and *fyuA*. The virulence factor *terC*, responsible for tellurite resistance, frequent in pathogenic bacteria, was the only virulence factor isolated in all 234 isolates. The *iss* gene, responsible for increased serum survival, was found in 191 (81.62%) of the 234 isolates. This gene is required for the synthesis of a capsule, necessary to protect the bacterium from complement proteins. The presence of capsule-expressing genes, like K-antigens, also seems to play an important role in extra-intestinal infections as 160 (68.38%) of all the 234 isolates carried one or more genes coding for a capsule. Interestingly, the gene coding for the K1-antigen (*kpsMII_K1*), a virulence factor that is strongly associated with neonatal meningitis, was present in 41 (17.52%) of the 234 isolates and only occurred in UPEC and ExPEC/UPEC isolates. ST95 and ST62 were found to be statistically associated (P < 0.001) with the occurrence of this gene. Both ST95 and ST62 are frequently isolated from cases of neonatal meningitis [14–16]. The genes *irp2* and *fyuA* are both responsible for iron uptake, an important virulence characteristic that 89.32% of the isolates harboured. Adherence-associated virulence factors were the most common: to this group belonged 47 different genes, of which 218 (93.16%) of the 234 isolates had at least one gene and 186 (79.49%) carried *irp2*. Except for the pathotype-associated genes, we found genes that were specifically present in the ExPEC and DEC groups. This is, for example, the case for virulence genes coding for toxins, which were found in 80.87% of ExPEC and UPEC isolates, but was not found in any of the three DECs.

In line with *Paramita et al*., we observed a correlation between ST and virulence genes, as strains within the same ST and serotype often harboured the same set of virulence genes [17]. We noticed that some genomic characteristics, like ST, are related to healthcare-acquired infections or their occurrence over time. In particular, ST131 occurred significantly (P = 0.0048) more often in the period after 2010 than before 2010. This finding is consistent with the literature, where ST131 was described for the first time in 2008 and has spread globally since then, becoming one of the most common multidrug-resistant *E. coli* lineages [3].

Different *E. coli* strains can spread both inside and outside a healthcare setting. In our study we describe four clusters, each consisting of two isolates, with identical (allelic difference of <5) strains based on cgMLST. For three of these cases, additional information was available (date of collection, admission department, place of residence, …) which weakly suggested that isolates within the same cluster could be associated with each other. In one of these cgMLST clusters (Figure 2), we found two ST131 strains, both isolated on the same day in two patients hospitalized for more than 48 hours in the same unit, suggesting healthcare-acquired transmission between the environment and patients or healthcare workers and patients. Nevertheless, cgMLST typing showed that most isolates are unrelated to each other, confirming the sporadic character of *E. coli* bacteraemia.

## Conclusion

This study provides insight into the prevalence of virulence factors in a large set of *E. coli* blood isolates from the UZ Brussel. It illustrates high diversity in virulence profiles and highlights the potential of DEC to carry virulence factors associated with extraintestinal infections, making it possible for unusual pathotypes to invade and survive in the bloodstream causing bacteraemia. Although these *E. coli* pathotypes are considered non-invasive, some of them cause bacteremia without being identified. We believe that the prevalence of diarrheagenic strains causing bacteremia is underreported, and that the increased access to WGS analysis will lead to a better understanding of the role of virulence factors in bacteraemic *E. coli* clinical isolates, in particular hybrid pathotypes. We also highlight the potential of WGS-based surveillance as an early warning system for emerging lineages, like *E. coli* ST131.

## Supplementary Material

Supplemental Material

## Data Availability

The datasets generated and/or analysed during the current study are available in the National Library of Medicine repository. BioProject: PRJNA854358
